# Aktualität der grundlegenden und determinierenden Bedeutung der chronisch-kritischen Extremitätenischämie sowie ihrer sich reetablierenden Behandlung mittels kruraler/pedaler Bypässe in Deutschland und in Sachsen-Anhalt

**DOI:** 10.1007/s00104-023-01933-7

**Published:** 2023-08-23

**Authors:** Udo Barth, M. Lehmann, F. Meyer, Z. Halloul

**Affiliations:** 1 Arbeitsbereich Gefäßchirurgie, Klinik für Allgemein‑, Gefäß- und Viszeralchirurgie, Helios Klinik Jerichower Land, August-Bebel-Straße 55a, 39288 Burg, Deutschland; 2 Arbeitsbereich Gefäßchirurgie, Klinik für Allgemein‑, Viszeral‑, Gefäß- und Transplantationschirurgie, Universitätsklinikum Magdeburg A. ö. R., Magdeburg, Deutschland Leipziger Str. 44, 39120

**Keywords:** Periphere arterielle Verschlusskrankheit, Revaskularisationsmaßnahmen, Majoramputationsrate, Risk stratification based on Wound, Ischemia, and Foot Infection, Global-Limb-Anatomic-Staging-System, Peripheral arterial occlusive disease, Revascularization measures, Major amputation rate, Risk stratification based on wound, ischemia, and foot infection, Global limb anatomic staging system

## Abstract

**Hintergrund:**

Aktuell ist eine Zunahme der schweren Stadien der peripheren arteriellen Verschlusskrankheit (pAVK) mit kritischer Ischämie zu verzeichnen. Dies scheint sowohl dem allgemeinen demographischen Wandel zu entsprechen als auch eine Folge der SARS-CoV-2(„severe acute respiratory syndrome coronavirus 2“)-Pandemie der letzten 3 Jahre zu sein. Das mittlerweile etablierte und akzeptierte interventionelle/endovaskuläre Vorgehen bei einer schweren Unterschenkel-pAVK in erfahrener Hand gilt nach wie vor als „First-line“-Therapie, jedoch erlebt aus eigener Sicht der krurale/pedale Venenbypass eine Renaissance.

**Material und Methoden:**

Kompakte narrative Übersicht über den aktuellen Stand der kruralen/pedalen Bypasschirurgie in Deutschland und Sachsen-Anhalt (SA), kombiniert mit selektiven Referenzen der aktuellen wissenschaftlich-medizinischen Literatur und eigenen klinischen Erfahrungen.

**Ergebnisse:**

Eine aktuelle Statistik der fallbezogenen DRG(„diagnosis related groups“)-Daten zeigt, dass insbesondere mit Auftreten der Corona-Pandemie ein Rückgang der stationären Fallzahlen von Patienten mit einem pAVK-Stadium IIB bundesweit und ebenfalls im Bundesland SA zu verzeichnen ist. Die schweren pAVK-Stadien blieben in den Fallzahlen annähernd gleich, in SA jedoch zunehmend. Die WIFI-Klassifikation bietet die Möglichkeit, über ein Punktesystem Aussagen über das Amputationsrisiko, Nutzen und Art der Revaskularisationsmaßnahme erstellen zu können. Verschlusslänge, Verschlusslokalisation der betroffenen Gefäße und Verkalkungsgrad finden Berücksichtigung im Global-Limb-Anatomic-Staging-System (GLASS) zur Beurteilung der Prognose. Die Auswertung der Fallpauschalenbezogenen Krankenhausstatistik von 2015 bis 2020 ergab eine konstante Anwendung der femorokruralen/-pedalen Bypasschirurgie in Deutschland sowie eine leichte Zunahme der Rekonstruktion mittels femorokruraler Bypässe in SA, die mit dem tendenziellen Fallzahlenanstieg der schweren pAVK zu korrelieren scheint.

**Schlussfolgerung:**

Zur Indikationsstellung für die Anlage eines kruralen/pedalen Bypasses sollte die parameterbasierte Objektivierung des Schweregrades der kritischen Extremitätenischämie mit einbezogen werden. Dafür eignen sich die WIFI-Klassifikation und GLASS, da hier auch eine relative Erfolgsprognose möglich ist. Die Behandlung der kritischen Extremitätenischämie mittels kruraler/pedaler Bypasschirurgie findet in Deutschland und SA nach wie vor eine konstante Anwendung.

## Abstract (en)

**Introduction:**

Currently, there is an increase in severe stages of peripheral arterial occlusive disease (PAOD) with critical ischemia. This seems to correspond to the general demographic change as well as a consequence of the severe acute respiratory syndrome coronavirus 2 (SARS-CoV-2) pandemic of the last 3 years. The now established and accepted interventional/endovascular approach for severe lower leg PAOD in experienced hands is still considered the first-line treatment but from the authorsʼ perspective crural/pedal venous bypass is experiencing a renaissance.

**Material and methods:**

Compact narrative review of the current state of crural/pedal bypass surgery in Germany and Saxony-Anhalt (SA) combined with selective references from the current scientific medical literature and own clinical experiences.

**Results:**

The current statistics of case-related diagnosis-related groups (DRG) data show that, especially with the occurrence of the corona pandemic, a decrease in inpatient case numbers of patients with PAOD stage IIB can be observed nationwide and also in SA. The severe PAOD stages have remained approximately the same in case numbers but increased in SA. The risk stratification based on the wound, ischemia and foot infection (WIFI) classification offers the possibility to be able to make statements about the risk of amputation, benefits and type of revascularization measures. The length of the occlusion, occlusion site of the affected vessels and degree of calcification are taken into account in the global limb anatomic staging system (GLASS) to assess the prognosis. The evaluation of the case-based hospital statistics from 2015 to 2020 showed a constant use of femorocrural/femoropedal bypass surgery in Germany as well as a slight increase in reconstruction using femorocrural bypasses in SA, which seems to correlate with the tendency for an increase in the number of cases of severe PAOD.

**Summary:**

Parameter-based objectification of the severity of critical limb ischemia should be included in the indications for placement of a crural/pedal bypass. The WIFI classification and GLASS are suitable for this purpose as a relative prognosis of success is also possible. The treatment of critical limb ischemia by crural/pedal bypass surgery continues to find a constant application in Germany and SA.

## Hintergrund

„Gefühlt“ kam es in den letzten 2 Jahren zu einem deutlichen Anstieg der Inzidenz der kritischen Extremitätenischämie gegenüber den geringeren Stadien der peripheren arteriellen Verschlusskrankheit (pAVK) im Einzugsgebiet der berichtenden Klinik. Im Jahr 2009 wurden in Deutschland nur 31,9 % der Patienten im Stadium III und IV und 68,8 % im Stadium II stationär therapiert [1]. In der einjährigen Aufbauphase der eigenen Gefäßchirurgie des Klinikums Jerichower Land in Burg betrug die Häufigkeit der Patienten mit einer kritischen Extremitätenischämie bereits 63,42 % der pAVK-Patienten. Dies lässt sich neben der demographischen Entwicklung und der damit verbundenen Zunahme der Herz- und Kreislauferkrankungen auch durch eine regionale gefäßmedizinische Unterversorgung erklären. Die Majoramputationsrate in Sachsen-Anhalt (SA), insbesondere im Jerichower Land ist in Deutschland mit am höchsten [2].

Entsprechend der Leitlinie soll bei der Behandlung der kritischen Extremitätenischämie nach individueller Abschätzung des Risiko-Nutzen-Verhältnisses der interventionellen Therapie zunächst der Vorzug gegeben werden. Grund dafür sind geringere Invasivität und niedrige Komplikationsraten. Dagegen sind häufigere Folgebehandlungen notwendig, zumeist Reinterventionen aufgrund von Restenosen und -okklusionen. Dadurch schien die Behandlung der kritischen Extremitätenischämie mittels kruraler/pedaler Bypässe in den Hintergrund getreten zu sein. Welchen Stellenwert die offene krurale und pedale Revaskularisation in den letzten Jahren eingenommen hat und welcher Tendenz sie unterliegt, soll im Folgenden erörtert werden. Eine Analyse der aktuellen gefäßmedizinischen Versorgungssituation in Deutschland und in einer strukturschwachen Region wie SA soll in die Erörterungen mit aufgenommen werden, um den Zusammenhang zwischen der Versorgungsrealität der chronisch-kritischen Ischämie und der Bedeutung der kruralen/pedalen Bypasschirurgie zu verdeutlichen. Des Weiteren werden die in Deutschland noch weniger bekannten Hilfsinstrumente zur Risiko- und Erfolgsstratifizierung wie die WIFI-Klassifikation (Risk stratification based on Wound, Ischemia and Foot Infection) und GLASS (Global-Limb-Anatomic-Staging-System) mit erläutert.

## Material und Methoden

Es wird eine narrative Kurzübersicht über den aktuellen Stand der Behandlung der kritischen Extremitätenischämie mittels kruraler/pedaler Bypasschirurgie in Deutschland und im Speziellen in Gegenüberstellung im Bundesland SA gegeben. Zudem erfolgte die Verwendung der fallpauschalenbezogenen Krankenhausstatistik zur pAVK und der codierten OPS(Operationen- und Prozedurenschlüssel)-Codes aus den Jahren 2015 bis 2020 in Deutschland und SA, die freundlicherweise vom Wissenschaftlichen Institut der AOK (WIdO) zur Verfügung gestellt wurden.

## Ergebnisse

### Aktuelle Daten zur pAVK in Deutschland und SA

**Figure 1:**
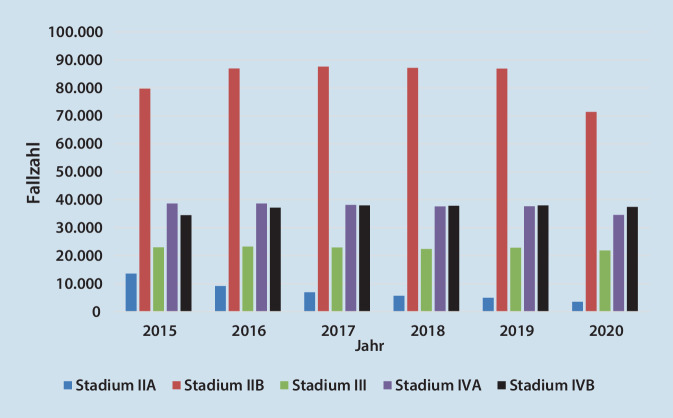

Eine aktuelle Statistik der fallbezogenen DRG(„diagnosis related groups“)-Daten zeigt, dass insbesondere mit Auftreten der Corona-Pandemie ein Rückgang der stationären Fallzahlen von Patienten mit einem pAVK-Stadium IIB bundesweit und ebenfalls in SA zu verzeichnen ist. Die schweren pAVK-Stadien blieben in den Fallzahlen annähernd gleich, tendenziell in SA jedoch zunehmend (Abb. 1**und** 2). Die Ursachen hierfür scheinen vielfältig zu sein, angefangen vom demographischen Wandel über die Pandemieproblematik bis hin zur nach wie vor verbesserungsbedürftigen ambulanten Begleitung der pAVK-Patienten. Rammos et al. zeigten in ihrer Studie, dass die Versorgung von pAVK-Patienten in Deutschland erschreckend mangelhaft ist. Nur 11 % der Patienten wurden im Jahr 2018 von einem Gefäßchirurgen und nur 8 % von einem Angiologen behandelt. Zudem erhielt nur die Hälfte der Patienten die leitliniengerechte Thrombozytenaggregations- und Statinmedikation [3].

**Figure 2:**
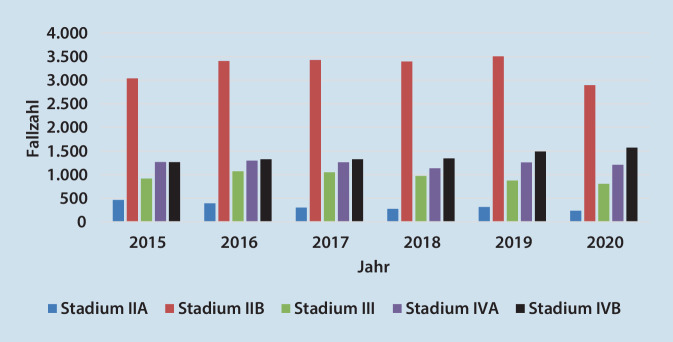

**Figure 3:**
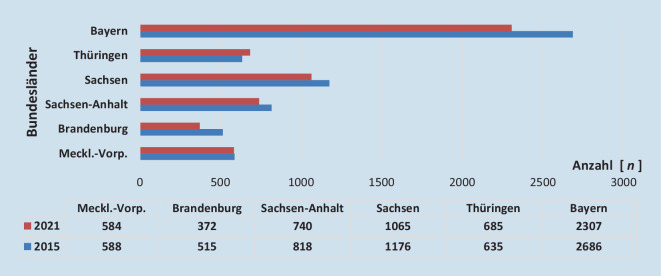

**Figure 4:**
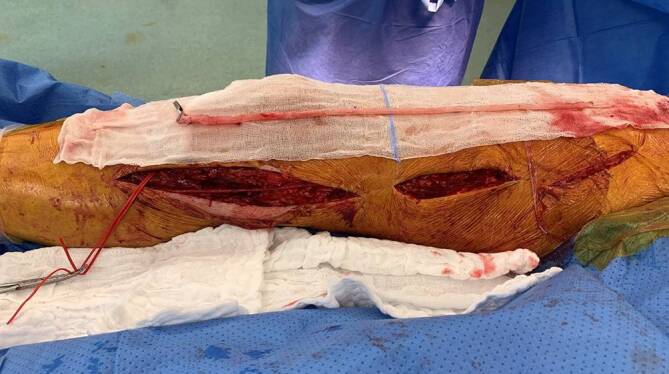

Nach wie vor rangiert SA bei der Majoramputationsrate in Deutschland sehr weit vorn. Bei einer Untersuchung der jährlichen bundesweiten Fallzahlen für die Jahre 2011 bis 2015 konnte gezeigt werden, dass überwiegend im Osten und Südosten höhere Amputationsraten bestehen. Insbesondere Kreise in Mecklenburg-Vorpommern, Brandenburg, Sachsen, SA, Thüringen und Bayern zeigten eine höhere „standardized mortality ratio“ (SMR) in mehreren Amputationshöhen. Diese auffälligen regionalen Unterschiede können durch die hohe altersadjustierte Prävalenz des Diabetes mellitus (mit-)begründet werden: z. B. 2015 für Ostdeutschland von 11,5 %, was sich mit den Regionen der Amputationsraten außerhalb des Konfidenzintervalls deckt, aber insbesondere abhängig ist von der Qualität der Versorgungsstrukturen [2]. Die Amputationsraten waren im Jahr 2021 in den o. g. Bundesländern bis auf Thüringen (Zunahme um 7,8 %) rückläufig. Der Rückgang der Amputationsraten in den Bundesländern Mecklenburg-Vorpommern (0,6 %), SA (9,5 %) und Sachsen (9,4 %) erscheint im Zeitraum von 6 Jahren sehr gering. Am deutlichsten fielen die Amputationsraten in Brandenburg (27,7 %) und Bayern (14,1 %; Abb. 3; [4]).

Die Notwendigkeit einer Verbesserung der gefäßchirurgischen Versorgung ländlicher und strukturschwacher Regionen zeigt sich einerseits in den Unterschieden der Intensität der vaskulären Versorgung, andererseits in der Erkenntnis, dass weiterhin in Deutschland ca. 40 % der Amputationen bei Patientinnen und Patienten mit einer kritischen Extremitätenischämie ohne einen zuvor unternommenen Revaskularisationsversuch durchgeführt werden [2].

### Offene krurale und pedale Rekonstruktionsverfahren

Die geringe Invasivität und technisch hohe Erfolgsrate der endovaskulären Therapie führte zur Etablierung der perkutanen transluminalen Angioplastie als „First-line“-Therapie in der aktuellen Leitlinie [5]. Dadurch ist die primäre krurale/pedale Bypassanlage ohne vorherige Intervention heutzutage sicherlich eine Seltenheit geworden. Dies könnte jedoch zu einer Selektion von endovaskulär austherapierten Patienten führen, aus der eine häufigere Nutzung weiter distal am Fuß gelegener Zielgefäße führt, die eine Verschlechterung der aktuellen Offenheitsraten pedaler Bypässe gegenüber früheren Studien erklären könnte [6]. Entsprechend dem Angiosom-Konzept sollte die Revaskularisation der Arterien erfolgen, welche das ischämisch betroffene Hautareal versorgen. In der Realität lässt die Auswahl an entsprechenden Zielgefäßen jedoch die Umsetzung des Angiosom-Konzeptes selten zu. Als entscheidend ist daher der Zustand des Anschlussgefäßes zu bewerten.

Insgesamt findet sich ein technisch ausreichendes und beherrschbares Repertoire an Rekonstruktionsmöglichkeiten. Grundsätzlich ist, soweit verfügbar, autologes Venenmaterial zu empfehlen. Bei Verfügbarkeit und entsprechendem Diameter (2,5–3 mm) ist die V. saphena magna prädestiniert (Abb. 4). Jedoch muss häufig bei schon bestehendem Verlust durch koronare Bypassoperationen auf Alternativen wie Armvenen, „spliced graft“, Kombinationen von autologem und alloplastischem Material, biosynthetische Prothesen oder komplett alloplastisches Material umgeschwenkt werden.

Bei Verwendung der autologen Vene sind verschiedene Anlageverfahren, „reversed“, „non-reversed“, in situ und „spliced“ mit ihren entsprechenden Vor- und Nachteilen möglich (Tab. 1, [7]).

**Table 1:** 

Konfiguration	Vorteile	Nachteile
„Reversed“	Keine Intimatrauma durch Vavulatomie	Kaliber-Mismatch
	Seitenäste sind ligiert	Venenklappen müssen zerstört werden
	Verlegung orthotop oder subkutan	
	Kontralaterale Venenentnahme möglich	
In situ	Kleines Präparationstrauma durch anastomosennahe Venenfreilegung	Venenklappen müssen zerstört werden
	Physiologisches Tapering	Bypassfrühverschluss bei unzureichender Klappendestruktion
		Ausbildung arteriovenöser Fisteln
„Non-reversed“	Physiologisches Tapering	Präparationstrauma
	Seitenäste abgesetzt	Valvulotomie notwendig
	Kontralaterale Venenentnahme möglich	Bypassfrühverschluss bei unzureichender Klappendestruktion
„Spliced“	Verwendbar, wenn keine ausreichend lange Vene vorhanden ist	Hoher präparatorischer Aufwand
		Mehr Reinterventionen zum Bypasserhalt
		Bypassoffenheit kürzer als bei VSM

*VSM* Vena saphena magna

### Was ist bei der Indikationsstellung zu beachten?

**Figure 5:**
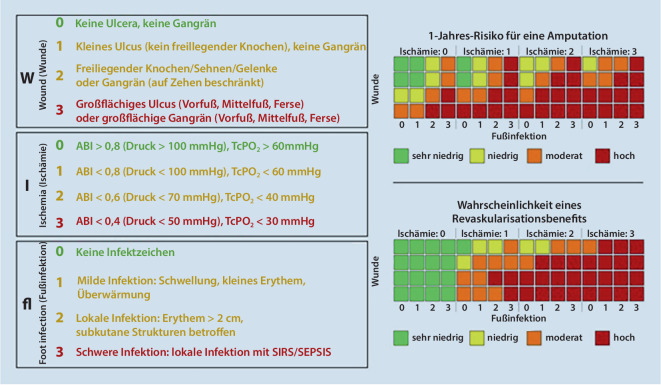

Nach wie vor sind die Erfolgsaussichten einer kruralen/pedalen Bypassanlage schwierig vorherzusagen. Daher sollte die Einschätzung des Schweregrades der chronisch-kritischen Ischämie in die Indikationsstellung mit einbezogen werden. Die Fontaine-Klassifikation unterscheidet nur zwischen Ruheschmerz und trophischen Störungen. Es sollten jedoch auch die Ausprägung der trophischen Störung, der Grad der Ischämie und die Schwere der begleitenden Infektion bei der Therapiestrategie und Prognosebeurteilung mit in die Betrachtungen einfließen. Hierzu kann die WIFI-Klassifikation (Risk stratification based on Wound, Ischemia and Foot Infection) angewendet werden. Für ein Punktesystem werden die Angaben zum klinischen Bild der trophischen Störungen (Ulkus, Gangrän), die Ausdehnung der trophischen Störungen (Zehen, Vorfuß, Rückfuß, Unterschenkel), Grad der Ischämie (Messung von „ancle-brachial index“ [ABI]/transkutaner Sauerstoffpartialdruck [tcpO_2_]) sowie der Grad der Infektion (lokal/systemisch, mild, moderat, schwer) benötigt. Nach Erstellung eines Scores können Aussagen über das Amputationsrisiko, Nutzen und Art der Revaskularisationsmaßnahme erstellt werden (Abb. 5, [8]).

Als weiteres Gradingsystem, welches die Verschlusslänge, Verschlusslokalisation sowie den Verkalkungsgrad der betroffenen Gefäße bei der Beurteilung eines Patienten mit einer kritischen Extremitätenischämie mit einbezieht, steht das Global-Limb-Anatomic-Staging-System (GLASS) zur Beurteilung der Prognose zur Verfügung. Dazu wird der zu behandelnden Extremität ein femoropoplitealer und ein infrapoplitealer Grad von 0–4 zugewiesen. Dieser korreliert mit einer leichten oder nichtsignifikanten Erkrankung der primären Zielarterie bis hin zu einem zunehmenden Schweregrad der Stenose und Krankheitslänge. Als primäre Zielarterie wird der optimale arterielle Pfad zur Wiederherstellung des Inline(pulsierenden)-Flusses zum Knöchel und zum Fuß definiert [9]. Um die inframalleolaren Arterien zu beschreiben, wird der Pedalmodifikator verwendet. Anhand des GLASS-Algorithmus erfolgt die Einteilung in 3 Stufen. Diese entsprechen einer niedrig-, mittel- oder hochkomplexen Erkrankung. Die Krankheitsmuster korrelieren mit dem unmittelbaren technischen Erfolg und der einjährigen Durchgängigkeit der Gliedmaßen bei einem endovaskulären Ansatz (Tab. 2, [10, 11]). Die Autoren selbst sehen GLASS als Grundlage für die klinische Praxis und Unterstützung zukünftiger klinischer Forschung der chronisch-kritischen Ischämie. Dennoch benötigt diese Einteilung eine prospektive Validierung in einer Vielzahl von Patientenpopulationen, sodass diese Einteilung noch Veränderungen und Anpassungen unterworfen sein wird [10].

**Table 2:** 

	Geschätzte PVI-Ergebnisse		
Stadium	Technisches Versagen (%)	1‑Jahres-LBP (%)	Anatomisches Befundmuster
I	< 10	>70	Kurze bis mittellange FP-Läsion und/oder kurze bis mittellange IP-Läsion; keine oder geringfügige popliteale Läsion
II	< 20	50–70	Mittellange bis lange FP-Läsion; ggf. mit Stenose der A. poplitea und/oder kurzer bis mittellanger IP-Läsion
III	> 20	< 50	Ausgedehnte FP- oder IP-Verschlüsse, allein oder in Kombination mit jeglicher Läsion im anderen Segment; CTO der A. poplitea

*CTO* „chronic total occlusion“ (chronischer Verschluss), *FP* femoropopliteal, *IP* infrapopliteal, *LBP* „limb-based patency“ (extremitätenbezogene Offenheitsrate), *PVI* periphere (endo-)vaskuläre Intervention

### Aktuelle Ergebnisse der kruralen/pedalen Bypasschirurgie

Die Studienergebnisse der letzten Jahre unterstreichen die guten Ergebnisse der kruralen/pedalen Bypasschirurgie, obwohl ein Vergleich der Studien aufgrund unterschiedlicher Qualitätskriterien schwierig ist. Suckow *et al*. berichteten 2013 über eine Untersuchung von 1227 Patientinnen und Patienten im Hinblick auf die 1‑Jahres-Offenheits- und Majoramputationsrate kruraler Bypässe mit Fokus auf den Vergleich von autologen V.-saphena-magna-Transplantaten und Prothesenbypässen. Die 1‑Jahres-Offenheitsraten für Vene und Prothese lagen bei 73 vs. 72 %, die Majoramputationsrate für Vene bei 13 % vs. 17 % beim Prothesenbypass. Als Erklärung für die kaum bestehenden Unterschiede in der primären Durchgängigkeit oder Blutungskomplikationen innerhalb des ersten Jahres nach der Operation und einem marginalen klinischen und statistischen Vorteil in Bezug auf die Rettung von Gliedmaßen und Überleben der Patientinnen und Patienten, die einen V.-saphena-Bypass erhielten, wurde von den Autoren die kurze Nachbeobachtungszeit von einem Jahr benannt [12]. Die meisten Erkenntnisse zu diesem Thema basieren auf Studien oder Metanalysen der 2010er Anfangsjahre.

Eine aktuelle Studie wurde von Abdul-Malak *et al*. publiziert. Sie untersuchten 2331 Patienten aus der Vascular Quality Initiative Infrainguinal Database von 2003 bis 2021 mit kruralen Bypässen mit paramalleolaren oder pedalen/plantaren Zielgefäßen. Sie ermittelten, dass die Prävalenz von „Unterschenkelbypässen“ zu distalen Zielen von 13,37 % aller „Lower-extremity-bypass“(LEB)-Eingriffe im Jahr 2003 auf 3,51 % im Jahr 2021 (*p* < 0,001) deutlich zurückgegangen ist. Schwerwiegende kardiale Ereignisse (MACE) traten zu 8,9 % auf, postoperative Wundinfektionen zu 3,6 % und die Majoramputationsrate der Patienten lag bei 16,8 % nach einem Jahr. Weitere Indikatoren waren die primäre Durchgängigkeit mit 50,56 ± 3,6 % und die sekundäre Durchgängigkeit mit 60,48 ± 4,12 %. Die Autoren schlossen, dass trotz geringerer Inanspruchnahme der offene chirurgische Bypass zu distalen Anschlussgefäßen am Knöchel nach wie vor eine praktikable Option für die Behandlung der infrapoplitealen kritischen Ischämie der Gliedmaßen mit akzeptabler Durchgängigkeit und amputationsfreien Überlebensraten nach 2 Jahren darstellt [13].

### Anwendung der kruralen/pedalen Bypasschirurgie in Deutschland und SA in den letzten Jahren

**Figure 6:**
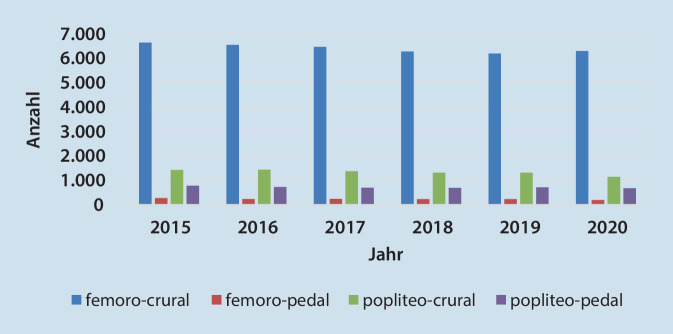

Die Anwendung der kruralen/pedalen Bypasschirurgie zur Behandlung der chronisch-kritischen Extremitätenischämie lässt sich anhand der Anzahl der kodierten OPS-Codes abschätzen. So ist für Deutschland insgesamt im Zeitraum 2015 bis 2020 die Anzahl für codierte femorokrurale, femoropedale, popliteokrurale und popliteopedale Bypässe leicht rückläufig. Einschränkend zur Wertung, muss man erwähnen, dass es sich um die reine Anzahl der eingegebenen OPS-Codes handelt, somit könnte es sich auch um mehrere Codes für ein und denselben Patienten handeln. Bei den kruralen Bypässen war ein Rückgang von ca. 8 % und bei den pedalen Bypässen von ca. 17 % von 2015 bis 2020 zu verzeichnen (Abb. 6). Den moderaten Rückgang der kruralen Bypassanlagen verzeichneten ebenfalls Kühnl *et al*. nach Auswertung der DRG-Codes von 2005 bis 2018 [14].

**Figure 7:**
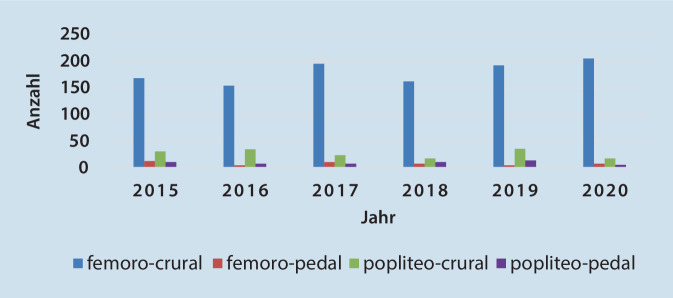

Im Bundesland SA stellt sich die Lage etwas eindrücklicher dar, obwohl die Menge der Daten aus statistischer Sicht sicherlich nur limitiert erscheint. Hier war jedoch ein Zuwachs der kruralen Bypassanlage um 11 % zu verzeichnen, während es bei der pedalen Bypassanlage zu einem Rückgang um 40 % kam (Abb. 7).

## Diskussion

Nach wie vor ist von einer hohen Anzahl an Patientinnen und Patienten mit einer kritischen Extremitätenischämie sowohl in Deutschland insgesamt als auch insbesondere in SA auszugehen, was zu persistierend hohen Kosten führt. Eine Verschärfung der Situation durch die anhaltende SARS-CoV-2(„severe acute respiratory syndrome coronavirus 2“)-Pandemie durch Aufschub und Verlagerung von Behandlungen und Operationen ist statistisch bei noch fehlenden Daten zwar zu vermuten, aber derzeit nicht zu beweisen. Die relativ konstanten Daten der chronisch-kritischen Extremitätenischämie in Deutschland sowie der Majoramputationen in strukturschwachen Bundesländern über den Zeitraum von 2015 bis 2021 unterstreichen die Bedeutung der kruralen/pedalen Bypasschirurgie in der Behandlung dieser fortgeschrittenen Gefäßerkrankung, was sich in der konstanten Zahl ihrer Anwendung widerspiegelt. Zumal die aktuelle wissenschaftliche Datenlage zu einer neuen Diskussion über den „Endovascular-first-Ansatz“ durch die Ergebnisse der Studie „Best Endovascular vs. Best Surgical Therapy in Patients With Critical Limb Ischemia (BEST-CLI)“ geführt hat, weil die Langzeitraten an sog. „major adverse limb events“ (MALE) und Tod bei Patienten mit guter Bypassvene gegenüber den endovaskulären Techniken besser und selbst bei weniger guten Venengrafts noch gleichwertig waren [15]. Somit ist von einer steigenden Bedeutung der kruralen/pedalen Bypasschirurgie in der Behandlung der CLI in den nächsten Jahren auszugehen.

Die krurale/pedale Bypasschirurgie hat trotz stetiger Verbesserung technischer Möglichkeiten der interventionellen Radiologie und endovaskulären Chirurgie ihren Stellenwert in der Behandlung der chronisch-kritischen Ischämie nicht verloren. Mit der WIFI-Klassifikation sowie GLASS kann bei der Entscheidungsfindung über den Behandlungsweg der Schweregrad der chronisch-kritischen Ischämie mit einbezogen und das Amputationsrisiko und der Nutzen der Revaskularisationsmaßnahme abgeschätzt werden. Jedoch sind zur Validierung dieser Klassifikationen weitere Studien notwendig. Hier gilt es, die Grundlagen durch Schaffung multizentrischer Datenbanken zu erweitern. Gerade in der deutschsprachigen Fachliteratur finden die aktuellen Klassifikationen und Bewertungssysteme kaum Erwähnung, was im Falle von GLASS an der Komplexität der Entscheidungsfindung liegen könnte.

Der Vergleich der Ergebnisse der offenen chirurgischen Therapie mit der endovaskulären Therapie bei der chronisch-kritischen Ischämie ist nach wie vor in der Diskussion, da hier eine zuverlässige Evidenz bislang fehlt. Bei einer aktuellen retrospektiven Bi-Center-Studie aus dem Jahr 2021 wurden zwischen dem 01.01.2012 und dem 01.01.2018 konsekutiv 1545 CLTI(„critical limb-threatening ischemia“)-Patienten mit femoropoplitealen GLASS-Grad-III/IV-Läsionen einbezogen, die einem Ersteingriff unterzogen worden waren. Die meisten Patientinnen und Patienten in der offen-chirurgischen Gruppe wurden mit Transplantaten unterhalb des Knies (87,23 %) vor allem aus autologer Vene (85,95 %) behandelt. Aus den Ergebnissen schlussfolgerten die Autorinnen und Autoren, dass die Bypassoperation bei femoropoplitealen Läsionen der Klassen III und IV bei Patientinnen und Patienten mit CLTI in Bezug auf die eher längerfristige primäre Durchgängigkeit und die Reinterventionsrate überlegen zu sein scheint, allerdings mit deutlich höheren postoperativen Komplikationsraten. Ein offener chirurgischer Zugang kann bei ausgewählten Patientinnen und Patienten als erste Wahl angesehen werden, insbesondere, wenn eine geeignete V. saphena magna verfügbar ist [16].

In diesem Zusammenhang erscheint die interdisziplinäre Zusammenarbeit zwischen Angiologinnen und Angiologen, interventionellen Radiologinnen und Radiologen sowie Gefäßchirurginnen und Gefäßchirurgen von eminenter Bedeutung, um ein optimales Therapieergebnis für die Patientinnen und Patienten zu erreichen. Sonst können Interessens- und Verteilungskonflikte zu Mehrfachuntersuchungen, Patientinnen- und Patienten-„Hopping“ und Ressourcenverschwendung führen. Die Gefäßchirurgin und der Gefäßchirurg erscheint als abstimmende(r) Koordinatorin/Koordinator einer interdisziplinären Behandlungsgruppe außerordentlich geeignet, da alle Stränge der Behandlung (konservativ, interventionell, operativ) bei ihr/ihm zusammenlaufen und von ihr/ihm beherrscht werden. Dies sollte dazu Anlass geben, das Fachgebiet Gefäßchirurgie auch auf der Ebene der Bundesländer zu stärken, z. B. durch Initiierung eines eigenen Lehrstuhls für Gefäßchirurgie an den ausstehenden Universitätskliniken. Diese könnten als Referenzzentren für Ausbildung, Forschung und Innovation fungieren und mit den gefäßchirurgischen Einheiten des Bundeslandes im Netzwerk eine zeitgerechte und adäquate Versorgung gefäßchirurgischer Patientinnen und Patienten gewährleisten. Gerade in einem so strukturschwachen Bundesland mit gering besiedelten ländlichen Regionen wie SA sollte eine gefäßchirurgische Universitätsmedizin als Leiteinrichtung unbedingt unterstützt und gefördert werden, da gerade in saisonalen Hochzeiten der kritischen Extremitätenischämie (Herbst, Winter und Frühjahr) die kleineren gefäßchirurgischen Einheiten aufgrund der kapazitiven Grenzen vor (teils kaum überwindliche) Herausforderungen gestellt werden und die Leiteinrichtung vermehrt frequentiert wird. Bei jedoch nur unterrepräsentierter Struktur von Personal- und Operationsressourcen kann die Leiteinrichtung dann nicht ihren eigentlichen Versorgungsauftrag in vollem Umfang gewährleisten. Somit kommt die Universitätsmedizin zunehmend in die Rolle, sektoren- und standortübergreifende Versorgungsketten zu organisieren oder auch verschiedene übersektorale Dienstleister und Netzwerke zu koordinieren [17].

Dass die Behandlung von Patientinnen und Patienten mit einer chronisch-kritischen Extremitätenischämie sehr hohe Kosten verursacht, steht außer Zweifel. So konnten Reinecke *et al*. bereits 2015 nachweisen, dass zwischen 2009 und 2011 56 % der Gesamtkosten für Gefäß-DRGs (336 Mio. €) auf CLI-Patientinnen und -Patienten entfielen [18]. Eine zusätzliche Herausforderung entsteht durch den anwachsenden Personalmangel in Pfleg- und Ärzteschaft, sodass hier dringend zeitnahe Maßnahmen gefordert sind, um eine entsprechende qualitativ hochwertige Versorgung der Patientinnen und Patienten zu gewährleisten. Dabei erscheint im ersten Schritt eine Verbesserung der ambulanten Versorgung von Patientinnen und Patienten mit einer pAVK gerade in strukturschwachen Regionen notwendig zu sein. Deshalb ist insbesondere die Zusammenarbeit der Hausärztinnen und -ärzte mit den gefäßchirurgischen Einheiten in den Kliniken zu stärken. Hier erscheint die „integrierte Versorgung“ (IV-Struktur) ein Mittel zu sein, um den Patientinnen und Patienten schneller und direkter eine gefäßchirurgische Behandlung zu ermöglichen. Der Gesetzgeber hat mit dem § 140a des Sozialgesetzbuches die Möglichkeit geschaffen, dass die Krankenkassen Verträge mit den in Absatz 3 genannten Leistungserbringern über eine besondere Versorgung abschließen können, die eine leistungssektorenübergreifende oder interdisziplinär fachübergreifende Versorgung sowie besondere Versorgungsaufträge unter Beteiligung der Leistungserbringer oder deren Gemeinschaften erlauben [19]. Durch die IV-Struktur wird zwar für die Patientinnen und Patienten die freie Arzt- und Klinikwahl durch geplante und vertraglich geregelte Abläufe eingeschränkt, jedoch wird eine klare Prozessstruktur und Patientinnen- und Patientensteuerung geschaffen, sodass die/der niedergelassene Ärztin/Arzt, das Krankenhaus, die Rehabilitationseinrichtungen und die Krankenkassen koordiniert und partnerschaftlich zusammenarbeiten können [20]. Auch sollte die Erkrankung der pAVK aufgrund ihrer deutlichen Relevanz für die Patientinnen und Patienten und das Gesundheitssystem durch mehr multimediale Aufklärung von Seiten der Krankenkassen und des Bundesgesundheitsministeriums in das Bewusstsein der Bevölkerung gerückt werden. Die von den gefäßmedizinischen Fachgesellschaften initiierten Mottoveranstaltungen zur pAVK sind dafür bei Weitem nicht ausreichend.

## Schlussfolgerung

Die krurale/pedale Bypasschirurgie hat sich auch am Beginn des neuen Jahrzehnts bei der Behandlung der chronisch-kritischen Extremitätenischämie bewährt. Die Ergebnisse aus kurz- und langfristiger Sicht können nach wie vor überzeugen. Die neu entwickelten Hilfsmittel zur Abschätzung des Schweregrades der CLI und Prognose sollten in der Praxis Anwendung finden, um deren Praktikabilität zu validieren und gegebenenfalls zu verbessern. Dies erfordert nach wie vor die schon lange geforderte Schaffung von Zentren regionaler und einrichtungsübergreifender Datenbanken zum Zwecke einer suffizienten klinischen (gefäßmedizinisch ausgerichteten) Versorgungsforschung. Das Aufkommen an Patienten mit einer CLI ist nach wie vor nicht unbeträchtlich hoch und sollte weiter sehr ernst genommen werden. Die hohen Kosten der Behandlung der fortgeschrittenen pAVK und die anhaltend hohe Anzahl von Majoramputationen in SA zeigen, dass die Gefäßmedizin im ambulanten und stationären Bereich noch nicht allerorts die erforderliche Aufmerksamkeit bekommt.
